# DNA Barcodes of Rosy Tetras and Allied Species (Characiformes: Characidae: *Hyphessobrycon*) from the Brazilian Amazon Basin

**DOI:** 10.1371/journal.pone.0098603

**Published:** 2014-05-30

**Authors:** Francis Paola Castro Paz, Jacqueline da Silva Batista, Jorge Ivan Rebelo Porto

**Affiliations:** 1 Facultad de Ciencias Biológicas, Universidad Ricardo Palma, Surco, Perú; 2 Laboratorio Temático de Biologia Molecular, Coordenação de Biodiversidade, Instituto Nacional de Pesquisas da Amazônia, Manaus-AM, Brazil; 3 Laboratório de Genética Animal, Coordenação de Biodiversidade, Instituto Nacional de Pesquisas da Amazônia, Manaus-AM, Brazil; Chang Gung University, Taiwan

## Abstract

DNA barcoding can be an effective tool for fast and accurate species-level identification based on sequencing of the mitochondrial cytochrome *c* oxidase subunit (COI) gene. The diversity of this fragment can be used to estimate the richness of the respective species. In this study, we explored the use of DNA barcoding in a group of ornamental freshwater fish of the genus *Hyphessobrycon*. We sequenced the COI from 10 species of *Hyphessobrycon* belonging to the “Rosy Tetra Clade” collected from the Amazon and Negro River basins and combined our results with published data. The average conspecific and congeneric Kimura 2-parameter distances were 2.3% and 19.3%, respectively. Six of the 10 species were easily distinguishable by DNA barcoding (*H. bentosi, H. copelandi*, *H. eques, H. epicharis*, *H. pulchrippinis*, and *H. sweglesi*), whereas the remaining species (*H. erythrostigma*, *H. pyrrhonotus, H. rosaceus* and *H. socolofi*) lacked reciprocal monophyly. Although the COI gene was not fully diagnostic, the discovery of distinct evolutionary units in certain *Hyphessobrycon* species under the same specific epithet as well as haplotype sharing between different species suggest that DNA barcoding is useful for species identification in this speciose genus.

## Introduction

Characidae is the largest family of the order Characiformes with approximately 163 genera and 1,057 valid species. This species richness represents approximately 52% of all species in the order. *Hyphessobrycon* is among the largest genera of Characidae and presently is placed in either “incertae sedis” or the “*Hemigrammus*” clade [1], [2].

Native to the Neotropics, *Hyphessobrycon* is widely distributed from southern Mexico to Argentina (Rio de la Plata) with the greatest species diversity found in the Amazon River basin [3], [4]. Approximately one-third of the *Hyphessobrycon* species are of commercial interest because they exhibit an attractive coloration pattern. Governmental regulations allow 45 Brazilian *Hyphessobrycon* species to be used for ornamental trade [5]. The Amazon basin is the primary fishing ground for South American ornamental fishes, including the *Hyphessobrycon* species [6], [7].

The morpho-anatomical characteristics used to distinguish *Hyphessobrycon* from other characids are not entirely diagnostic. These characteristics include the lack of scales on the caudal fin, an incomplete lateral line, more than one row of pre-ventral scales, the presence of an adipose fin, two series of pre-maxillary teeth with the inner series containing five teeth, a lack of ventral contact between the second suborbital and the preopercle, and few maxillary teeth [6], [8]–[10].

Based on their color patterns, *Hyphessobrycon* species have been divided into six admittedly artificial species groups: **(a)** species without black markings on the body, **(b)** species with one or two humeral spot(s), **(c)** species with a caudal spot, **(d)** species with both humeral and caudal spots, **(e)** species with a longitudinal pattern, usually a band uniting the humeral and caudal spots, and **(f)** species with a black spot on the dorsal fin, including two subgroups (*bentosi* and *compressus*) [6].

Because the primary grouping of this genus relies on similarities in the pigmentation patterns, it is difficult to identify characteristics that are useful to formulate hypotheses on the relationships among the species; therefore, some researchers do not accept or follow this classification. Conversely, other groups [11], [12] have concluded that the pigmentation patterns of *Hyphessobrycon* might be useful for ordering the complex systematic relationships within the genus.

Despite being considered the most speciose genus in Characidae, the inter- and intraspecific relationships within *Hyphessobrycon* remain largely unresolved. According to recent phylogenetic hypotheses on Characidae, *Hyphessobrycon* is clearly polyphyletic [7], [13]–[17]. However, ongoing studies and unpublished phylogenetic hypotheses on *Hyphessobrycon* have revealed that at least two groups are monophyletic: 1) the “true” *Hyphessobrycon*, which partially encompasses the Rosy Tetra clade, and 2) the “heterorhabdus” clade [Carvalho, pers. comm. Universidade Estadual Paulista; García-Alzate, pers. comm Universidad del Atlántico].

Morphological characteristics are not always sufficient to identify certain species, especially when their phenotypes are diverse. In addition, the use of species identification keys, often effective only at a certain stage of life, does not always allow for the correct diagnosis of a taxon. Therefore, DNA has been used as an alternative tool for the diagnosis of species with or without an integrative taxonomic approach [18]–[20].

DNA barcoding is a taxonomic method that uses a standardized short fragment of DNA to identify previously known species and facilitate the rapid recognition of new species [18]. The cytochrome *c* oxidase subunit I (COI) gene is most commonly used, but the use of other loci has been proposed [21]. In DNA barcoding, there are two main underlying assumptions: the reciprocal monophyly of species and an intraspecific divergence less than interspecific divergence [22]. DNA barcode-based identification is effective in discriminating species. However, the error rates can be high when there are no reference data, when samples do not reflect a species entire range, and when data for closely related species are unavailable [23], [24].

DNA barcoding has been used for Neotropical ichthyofaunal surveys of specific rivers [25] or regions [26]–[28] and to study specific taxa [29], describe new species [30], identify cryptic species [31], and identify commercial products [32]. This study aimed to improve the accuracy of identification of the Rosy Tetras and allied species of *Hyphessobrycon* and investigate whether the COI gene is effective for the efficient DNA-based identification of *Hyphessobrycon* congeners.

## Materials and Methods

### Ethics Statement

This survey was conducted in strict accordance with the recommendations of the National Council for Control of Animal Experimentation and Federal Board of Veterinary Medicine. The protocol was approved by the Committee on the Ethical Use of Animals (040/2012) of the Instituto Nacional de Pesquisas da Amazônia (INPA). All specimens for this study were collected in accordance with Brazilian laws under a permanent scientific collection license approved by the Brazilian Institute of Environment and Renewable Natural Resources (IBAMA) through the System Authorization and Information on Biodiversity (SISBIO #11489-1and 25890-1).

### Taxon sampling

**Figure 1:**
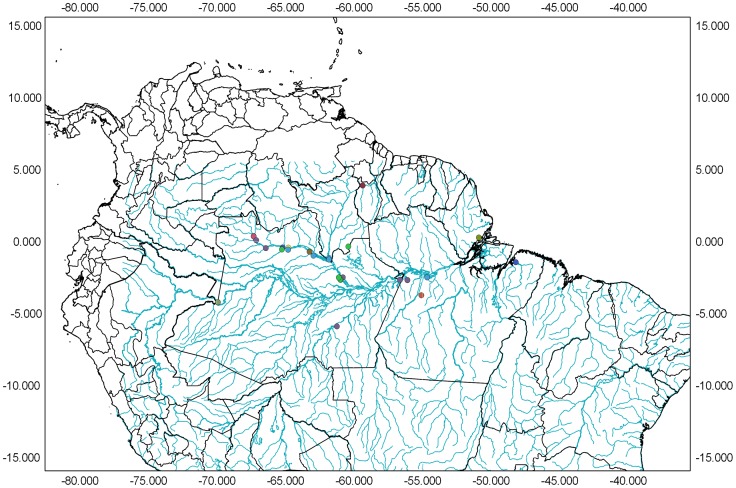
Map showing the sample distribution in the Amazon Basin.

We collected 158 fishes belonging to 10 species at 28 different sites located throughout the Amazon and Negro River basins (Figure 1 and Table S1). Whole specimens (adult fish and juveniles) were collected for genetic analysis and storage as voucher specimens. All of the specimens were anesthetized by immersion in Eugenol and preserved in 96% ethanol. Morphological identification was performed by taxonomists and confirmed using published and unpublished identification keys. After identification, morphological vouchers were deposited in the Zoological Collection at the National Institute for Amazonian Research (INPA). Specimen data, including the geospatial coordinates of the collection sites and other relevant details, are available in the BOLD database (http://v3.boldsystems.org/) under the project “DNA Barcoding of *Hyphessobrycon* – HYP”.

### DNA isolation, amplification and sequencing

DNA was isolated from the muscle tissue of each specimen using two methods: a DNeasy Tissue Kit (Qiagen) according to the manufacturer's instructions or a modified phenol-chloroform protocol described by Sambrook *et al.* [33]. Subsequently, the 650-bp barcode region of the mitochondrial COI gene (hereafter referred to as COI-5P) was amplified using the primers LCO1490 and HCO2198 [34].

The polymerase chain reaction (PCR) was performed in a total volume of 15 µl containing 10–50 ng of DNA template, 1X buffer (750 mM Tris-HCl, pH 8.8, 200 mM (NH_4_)_2_SO_4_, 0.1% (v/v) Tween 20), 1 unit of *Taq* polymerase (Fermentas -Thermo Scientific), 0.2 mM dNTPs, 0.2 µM of each primer, 2 mM MgCl_2_ and ultrapure water. The PCR program was as follows: 95°C for 2 min; 35 cycles at 93°C for 30 s, 41°C for 40 s and 72°C for 1 min and 20 s; and a final extension at 72°C for 7 min.

All PCR products were purified using a GFX kit (GE Healthcare) according to the manufacturer's protocols, and bi-directional sequencing was performed using an ABI BigDye Terminator v.3.1 Cycle Sequencing Ready Reaction Kit and an ABI 3130xl DNA Analyzer (Applied Biosystems, Inc.) according to the manufacturer's instructions. The cycle sequencing conditions included an initial denaturation step of 1 min at 96°C followed by 15 cycles of 96°C for 10 s, 50°C for 10 s, and 60°C for 1 min and 15 s followed by 5 cycles at 96°C for 10 s, 50°C for 10 s and 60°C for 1 min and 30 s and a final step of 5 cycles at 96°C for 10 s, 50°C for 10 s and 60°C for 2 min.

### Data analysis

The forward and reverse COI-5P sequences were aligned using the ClustalW Multiple Alignment tool in the software BioEdit v7.0.1 [35] and edited manually. The COI nucleotide sequences were translated to amino acid sequences to detect insertions, deletions, or stop codons. The sequences were aligned using the tools available on BOLD v3.0 (http://v3.boldsystems.org). Genetic distances between specimens were calculated with the “Distance Summary” command implemented by BOLD. The genetic distances were calculated using the Kimura 2-parameter (K2P) distance model [36]. Neighbor-joining [37] analyses of K2P distances was performed using the MEGA v5.0 [38] software to provide a graphical representation of the pattern of divergence among the species. Node support was evaluated based on 1000 bootstrap replicates. A maximum-likelihood analysis was performed using the program PhyML [39] with the HKY85 substitution model, which was the optimum model calculated using jModeltest [40] specifications.

We supplemented the data gathered in this study with the following sequences from the GenBank and BOLD databases: *Hyphessobrycon anisitsi* (FJ749040-GBGCA516-10), *Hyphessobrycon bifasciatus* (HM064999) *Hyphessobrycon erythrostigma* (FJ749055-GBGCA501), *Hyphessobrycon eques* (FJ749057-GBGCA499-10 and JF752341-ANGBF1897-12), *Hyphessobrycon herbertaxelrodi* (FJ749053-gbgca503-10), *Hyphessobrycon megalopterus* (FJ749058-GBGCA498-10), and *Hyphessobrycon santae* (HM405129). Sequences from *Hyphessobrycon pulchripinnis* and *Moenkhausia hemigrammoides* were also included as outgroups to the Rosy Tetra clade.

## Results

We sequenced the COI gene in 158 specimens; the number of specimens per species varied from 1 to 36 with an average of 15 (Table S1). The ten *Hyphessobrycon* species examined in this study were collected in the Negro River basin (*H*. *bentosi*, *H*. *copelandi*, *H*. *epicharis*, *H*. *pyrrhonotus*, *H*. *rosaceus*, *H*. *socolofi*, and *H*. *sweglesi*) and the Amazon River Basin (*H*. *copelandi*, *H*. *eques*, *H*. *erythrostigma*, and *H*. *pulchripinnis*). Additionally, we sequenced *Moenkhausia hemigrammoides* from Guyana as an outgroup (Figure 1, Table S1). We performed taxonomic identification at the species level for all 158 individuals based on the identification key (morphology). We found that 155 specimens belonged to the genus *Hyphessobrycon* and that three belonged to the genus *Moenkhausia*.

DNA sequencing yielded 650 COI-5P barcodes, and no stop codons, deletions, or insertions were observed. Nucleotide composition analysis revealed that the mean frequencies for the complete data set were 19.6% G, 27.3% C, 22.5% A, and 30.0% T.

Almost all species had mean conspecific divergence values below 2%, with the exceptions of *H*. *copelandi* (2.2%), *H*. *rosaceus* (8.9%), and *H*. *socolofi* (4.3%). The absence of a “barcode gap” (when the minimum between-species sequence distance is less than the maximum within-species distance) was evident between some closely related species-pairs (*H*.*bentosi* x *H*. *socolofi* – 0.6%, *H*. *erythrostigma* x *H*. *pyrrhonotus* – 0.4%, *H*. *pyrrhonotus* x *H*. *socolofi* – 0%) (Table 1). The mean conspecific divergence found in *Hyphessobrycon* was 2.3%, and the mean congeneric divergence was 19.3% (Table 1).

**Table 1:** The mean and maximum intra-specific values compared to the nearest neighbor distance in *Hyphessobrycon* species from the Brazilian Amazon basin.

Species	Mean Intra-Sp	Max Intra-Sp	Nearest Species	Distance to NN
*Hyphessobrycon bentosi*	0.12	0.31	*Hyphessobrycon socolofi*	**0.62**
*Hyphessobrycon copelandi*	2.28	6.05	*Hyphessobrycon eques*	10.45
*Hyphessobrycon epicharis*	1.76	4.66	*Hyphessobrycon sweglesi*	3.33
*Hyphessobrycon eques*	0.11	0.16	*Hyphessobrycon copelandi*	10.45
*Hyphessobrycon erythrostigma*	1.88	3.99	*Hyphessobrycon pyrrhonotus*	**0.46**
*Hyphessobrycon pulchripinnis*	N/A	N/A	*Hyphessobrycon rosaceus*	22.55
*Hyphessobrycon pyrrhonotus*	1.82	3.66	*Hyphessobrycon socolofi*	**0**
*Hyphessobrycon rosaceus*	8.92	22.28	*Hyphessobrycon sweglesi*	7.06
*Hyphessobrycon socolofi*	4.35	11.60	*Hyphessobrycon pyrrhonotus*	**0**
*Hyphessobrycon sweglesi*	0	0	*Hyphessobrycon epicharis*	3.33

N/A corresponds to a singleton for intra-specific values. Bolded distances correspond to the nearest neighbor (NN) that is less than 2% divergent.

**Figure 2:**
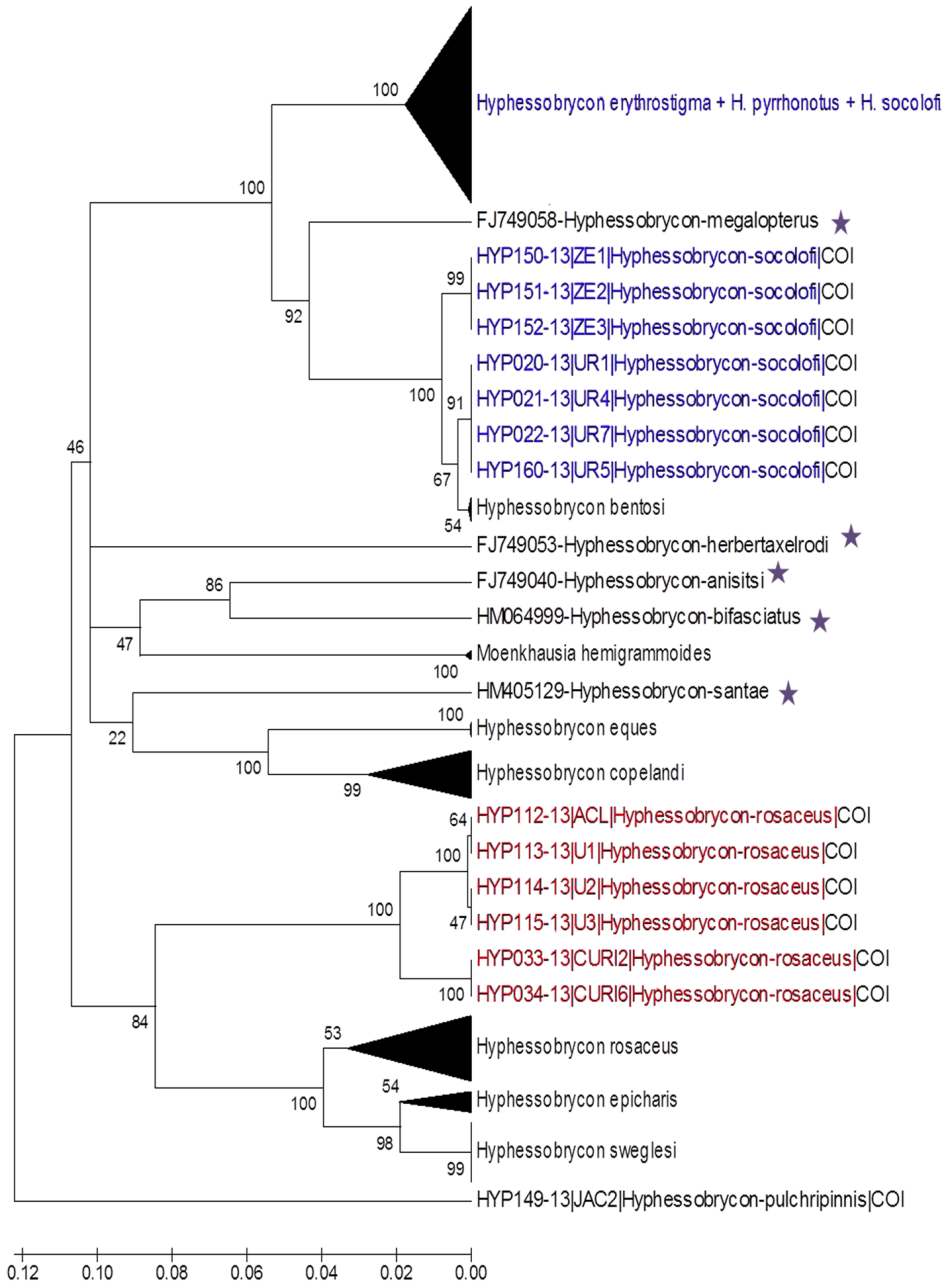
Neighbor-joining (NJ) tree of select *Hyphessobrycon* taxa showing *H. rosaceus* (marked in red) and *H. socolofi* (marked in blue) as probable evolutionary units. Node values are the bootstrap test results (1,000 pseudo-replicates). The stars indicate species for which sequences were obtained from the GenBank database.

**Figure 3:**
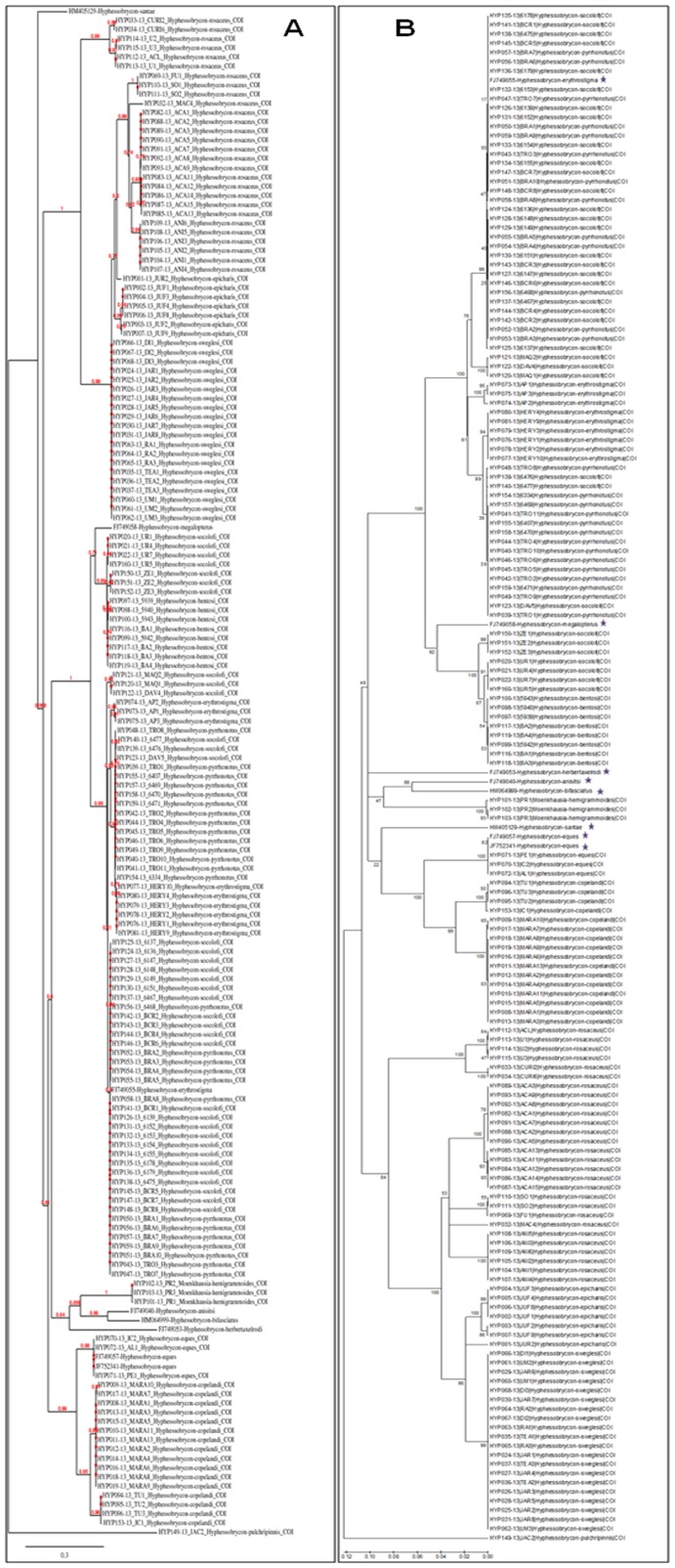
Maximum likelihood (A) and neighbor-joining (B) COI trees of all *Hyphessobrycon* species sequenced to date including the outgroup *Moenkhausia hemigrammoides*.

The K2P neighbor-joining tree showed that most species in this study formed reciprocally monophyletic groups. The following nominal *Hyphessobrycon* species were readily distinguishable using the DNA barcoding approach: *H*. *anisitsi*, *H*. *bifasciatus*, *H. bentosi*, *H*. *eques*, *H*. *epicharis*, *H*. *herbertaxelrodi*, *H*. *megalopterus*, *H*. *pulchripinnis*, *H*. *santae*, and *H*. *sweglesi*. However, four species could not be accurately identified: *Hyphessobrycon erythrostigma*, *H*. *pyrrhonotus, H*. *socolofi*, and *H. rosaceus* (Figure 2). The outgroup *Moenkhausia hemigrammoides* was found to be paraphyletic. The ML and NJ methods yielded nearly identical tree topologies (Figure 3, File S2).

Two *Hyphessobrycon* species, *H*. *rosaceus* and *H*. *socolofi*, were paraphyletic and yielded the two highest observed maximum intraspecific genetic distances (22.2% and 11.6%, respectively). *H*. *rosaceus* consisted of two distinct groups: 1) four specimens from the Amazon River and two from the Upper Negro River; and 2) 22 specimens distributed along the Upper and Lower Negro River and the Amazon River basin that formed a clade with *H*. *epicharis* and *H*. *sweglesi*. However, *H*. *socolofi* constituted two distinct groups: 1) 29 specimens that clustered with *H*. *erythrostigma* and *H*. *pyrrhonotus*; and 2) seven specimens (four from the Urubaxi River in the Middle Negro River basin and three from Benevides, Eastern Amazon) that clustered with *H*. *bentosi* (from the Middle Negro river) (Figure 2, File S2, Table 1, Table S1).

**Figure 4:**
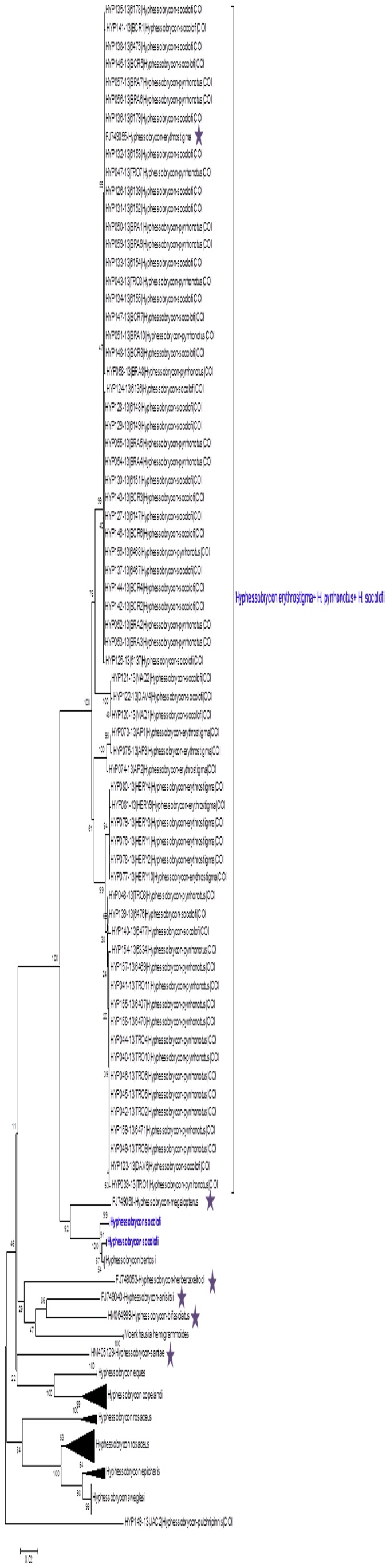
Neighbor-joining (NJ) tree of select *Hyphessobrycon* taxa showing the non-monophyletism of *Hyphessobrycon erythrostigma*, *H*. *pyrrhonotus* and *H*. *socolofi* (marked in blue) and the possible geographic segregation of *H*. *socolofi*. Node values are the bootstrap test results (1,000 pseudo-replicates). Stars indicate species for which sequences were obtained from the GenBank database.

A large clade consisting of specimens of *H*. *erythrostigma*, *H*. *pyrrhonotus*, and *H*. *socolofi* was observed. In this clade, haplotype-sharing events between *H*. *socolofi* and *H*. *pyrrhonotus* were detected. An apparent geographical segregation between specimens of *H*. *pyrrhonotus* was observed, as evidenced by a distinct sub-clade consisting exclusively of specimens from the Urubaxi river at the right bank of the Negro River (*n* = 9) that differed from a sub-clade of specimens from the Daraá river at the left bank of the Negro river (*n* = 10) and specimens from the Urubaxi river (*n* = 2) (Figure 4, File S1).

Distinct lineages were also observed in *H*. *copelandi*. The first group includes 12 specimens from the Marauiá River (upper Negro River). The second group includes three specimens from the Urumutum River (Tabatinga - Western Amazon) and one from the Maicá Lake (Santarém city - Eastern Amazon) (File S1).

## Discussion

The isolated application of morphological or DNA characteristics for species identification has been criticized and has various caveats, especially when very few individuals are sampled per species or only a small fraction of the global species richness is considered [23], [24]. Our study on 158 specimens belonging to 10 species of *Hyphessobrycon* showed that six species (60%) were easily distinguishable by DNA barcoding: *H. bentosi*, *H. copelandi*, *H*. *eques*, *H*. *epicharis*, *H*. *pulchripinnis*, and *H*. *sweglesi*. Three species (*Hyphessobrycon erythrostigma*, *H*. *pyrrhonotus*, and *H*. *socolofi*) could not be delineated based on COI gene sequences because each lacked reciprocal monophyly, and two species (*H. socolofi* and *H*. *rosaceus*) might possess hidden diversity because they consisted of two clades (Figure 2, File S1).

Overall, studies on North American and Neotropical freshwater ichthyofauna have revealed that the mean congeneric and conspecific genetic distances are usually approximately 6.8% and 0.7%, respectively [25], [27], [28], [41]–[43]. The mean genetic divergence observed in *Hyphessobrycon* (19.3%) was three times higher than the aforementioned genetic distances. One possible explanation could be the higher rates of evolution or ancient divergences in Hyphessobrycon.

In the clade that includes *H*. *bentosi, H. erythrostigma, H. pyrrhonotus*, and *H. socolofi*, a group of species with few morphological divergences [7], we observed the absence of a barcode gap (Table 1, Figure 4). In the DNA Barcode literature, the absence of barcode gaps and the paraphyly/polyphyly of conspecific DNA sequences have been explained as results of incomplete lineage sorting [44]. The minimum pairwise differences observed between the species forming this clade were below 0.6%. The comparatively low sequence divergence observed among these species may occur because they most likely are recently diverged species and present incomplete lineage sorting. Apparently, these species have not had sufficient time to accumulate mutations in the COI gene due to recent speciation. Thus, DNA barcoding would fail to identify them.

Additionally, if we examine the present distribution pattern of the endemic species *H. socolofi* and *H. pyrrhonotus* in the Negro River Basin, we cannot rule out an event of peripatric speciation in which a small population, at the extreme edge of the species range, became separated into a different species. In this particular case, *H. socolofi* possesses a distribution pattern that encompasses the entire Negro River Basin, whereas *H. pyrrhonotus* shows a more restricted distribution pattern by occurring in only in three Negro River tributaries (Daraá, Ererê and Urubaxi).

Furthermore, we detected evidence for haplotype sharing between *H*. *pyrrhonotus* and *H*. *socolofi*. Freshwater fish are among the groups of animals with the most frequent interspecific haplotype sharing, reported at 2% in Australian marine fishes, 8% in Canadian freshwater fish, 4% in Cuban freshwater fishes, 10% in North American freshwater fish and 11.4% in Nigeria freshwater fish [41]–[43], [45], [46]. The haplotype sharing observed in these studies appears to have resulted from hybridization, incomplete lineage sorting, inadequate taxonomy, and erroneous identification. In *Hyphessobrycon*, the detection of interspecific haplotype sharing in two of ten analyzed species leads us to infer that the likely explanations are incomplete lineage sorting or hybridization. In contrast, poor taxonomy is a likely cause of this pattern.

After publication of the results of Ward and colleagues [47], several research groups used a threshold of 2% for conspecific genetic divergence in fishes. In *Hyphessobrycon,* most of the studied species were within the threshold of 2% for conspecific genetic distance. However, the observed maximum conspecific divergence in six out of the 10 species showed a remarkably high level of intraspecific genetic distances (3.6%–22.2%). These values are more likely to be congeneric than conspecific. Considering that *Hyphessobrycon* species are not readily distinguishable by their external morphology, we suggest that there are cryptic species for at least some of these six species. On average, the conspecific genetic divergence detected in previous fish surveys is lower than our observations using *Hyphessobrycon* (e.g., Australian marine fishes (0.3%), Canadian fishes (0.2%), North American fishes (0.7%), African fishes (0.1%), Persian fishes (0.1%), and Neotropical fishes (1.3%)) [28]–[41], [43], [46], [48]. Obviously, if the hidden species are properly identified and taxonomically validated then the conspecific genetic divergence in *Hyphessobrycon* will be decreased.

There have been several reports of DNA barcodes being used to discriminate cryptic fish species e.g., [49]–[51]. Usually, cryptic species complexes cannot be easily identified based on classical morphology despite high levels of conspecific genetic distance [52]. This appears to be true for *H*. *socolofi* and *H*. *rosaceus*. Although groups of specimens within *H*. *socolofi* and *H*. *rosaceus* were indistinguishable using morphological methods, molecular characteristics unequivocally separated these groups. In the Neotropical fish species, more than 20 cases of possible cryptic speciation were detected when the conspecific divergence was greater than 2% [28], [53].

The Amazon basin has the most diverse freshwater fish fauna in the world [54], [55]. The large number of described *Hyphessobrycon* species (131 spp.) and the new species described every year reveal the astonishing species richness of the genus. Within in the past 10 years, 35 new species have been described [2]. Several factors including the unique geomorphological features of the Neotropics and preservation of the extraordinary species richness characterize the modern Neotropical ichthyofauna [56].

Historically, *Hyphessobrycon* species have been described based on morphological characteristics, including similarities in the pigmentation patterns, using a low number of individuals per species. DNA barcoding in *Hyphessobrycon* can be used to discriminate species and identify new ones and reveals that it is not always possible to differentiate good species based solely on their morphology. Because our study revealed likely cryptic speciation in *Hyphessobrycon,* we recommend the use of DNA barcodes for future descriptions of new species to increase our understanding of this speciose genus.

## Supporting Information

Table S1 **List of the 158 analyzed specimens.**
(XLS)

File S1 **Neighbor-joining (NJ) tree of the genus** ***Hyphessobrycon*** **.** The NJ tree of the COI sequences of 158 specimens calculated using the Kimura 2-parameter distance model. Node values are the bootstrap test results (1,000 pseudo-replicates). Stars indicate species for which sequences were obtained from the GenBank database.
(TIF)

File S2 **Maximum likelihood phylogenetic tree of 155 COI barcodes from 10 species of** ***Hyphessobrycon*** **.** *Hyphessobrycon pulchripinnis* and *Moenkhausia hemigrammoides* were used as outgroups.
(PDF)
