# Supplementary figures and images for: DNA Barcodes of Rosy Tetras and Allied Species (Characiformes: Characidae: Hyphessobrycon) from the Brazilian Amazon Basin
Source: PLoS One. 2014 May 30;9(5):e98603. doi: 10.1371/journal.pone.0098603 (PMC4039478; doi:10.1371/journal.pone.0098603)

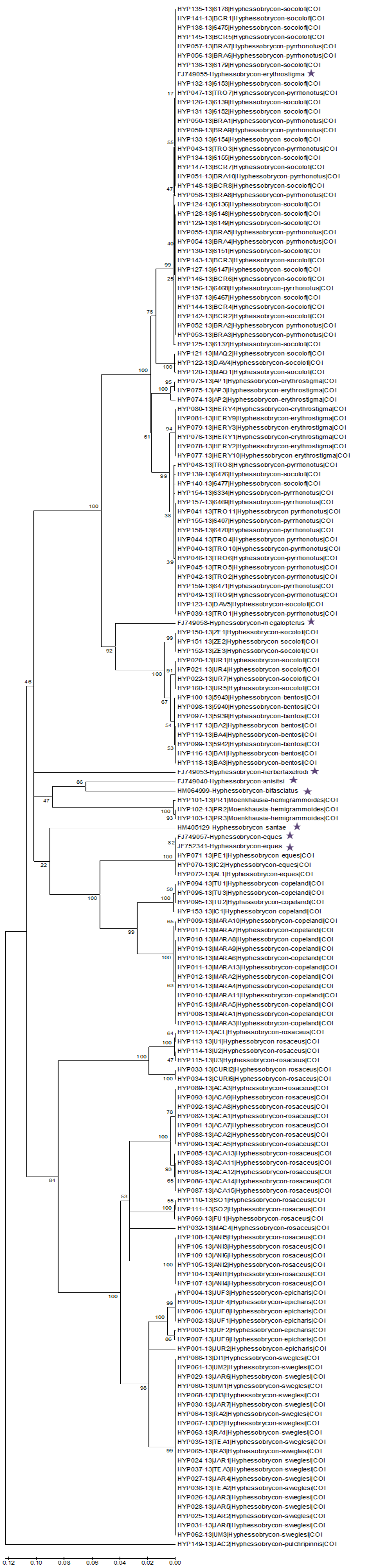

Supplement: File S1 — Neighbor-joining (NJ) tree of the genus Hyphessobrycon . The NJ tree of the COI sequences of 158 specimens calculated using the Kimura 2-parameter distance model. Node values are the bootstrap test results (1,000 pseudo-replicates). Stars indicate species for which sequences were obtained from the GenBank database. (TIF) [file pone.0098603.s002.tif]

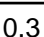

Supplement: File S2 — Maximum likelihood phylogenetic tree of 155 COI barcodes from 10 species of Hyphessobrycon . Hyphessobrycon pulchripinnis and Moenkhausia hemigrammoides were used as outgroups. (PDF) [file pone.0098603.s003.pdf]
